# Animal Welfare in Predator Control: Lessons from Land and Sea. How the Management of Terrestrial and Marine Mammals Impacts Wild Animal Welfare in Human–Wildlife Conflict Scenarios in Europe

**DOI:** 10.3390/ani10020218

**Published:** 2020-01-29

**Authors:** Laetitia Nunny

**Affiliations:** Wild Animal Welfare, La Garriga, 08003 Barcelona, Spain; laetitia.nunny@me.com

**Keywords:** animal welfare, human–wildlife conflict, pest, predator, welfare assessment, welfare domains, welfare impact, wildlife

## Abstract

**Simple Summary:**

Both marine and terrestrial mammal predators come into conflict with humans in Europe and yet their situations are rarely compared. Areas of conflict include the predation of livestock and farmed fish, and also perceived competition for wild prey (for example wolves competing with hunters for deer and seals competing with fishermen for salmon). A lethal method (shooting) and non-lethal methods of conflict reduction used for terrestrial large carnivores (e.g., bear, wolf, wolverine, lynx) and marine mammals (seals) are discussed and their potential impacts on predator welfare are considered. The importance of carrying out an animal welfare assessment when choosing a control method is emphasized along with possible assessment methods. Recommendations for future work are also made.

**Abstract:**

The control of predators, on land and in the sea, is a complex topic. Both marine and terrestrial mammal predators come into conflict with humans in Europe in many ways and yet their situations are rarely compared. Areas of conflict include the predation of livestock and farmed fish, and the perceived competition for wild prey (for example wolves competing with hunters for deer and seals competing with fishermen for salmon). A lethal method (shooting) and non-lethal methods of conflict reduction (including enclosures, guarding, and aversion) used for terrestrial large carnivores (e.g., bear, wolf, wolverine, lynx) and marine mammals (seals) are discussed. Control measures tend to be species- and habitat-specific, although shooting is a widely used method. Potential impacts on predator welfare are described and welfare assessments which have been developed for other wildlife control scenarios, e.g., control of introduced species, are considered for their potential use in assessing predator control. Such assessments should be applied before control methods are chosen so that decisions prioritizing animal welfare can be made. Further work needs to be carried out to achieve appropriate and widely-accepted animal welfare assessment approaches and these should be included in predator management planning. Future research should include further sharing of approaches and information between terrestrial and marine specialists to help ensure that animal welfare is prioritized.

## 1. Introduction

Wild animals come into conflict with humans in Europe in a variety of circumstances and, although both marine and terrestrial mammalian predators are involved in such interactions, their situations are rarely compared. Studies looking at human–wildlife conflict have historically focused more on conflict with terrestrial, rather than marine, predators [1]. The predation of livestock and other animals reared for human use, such as farmed fish, is a key area of conflict and predators are managed in a variety of ways to prevent them from injuring and killing these animals [2,3]. Other areas of conflict include the perceived competition for wild prey (for example wolves competing with hunters for deer and seals competing with fishermen for salmon) [4,5], the predation of endangered prey which wildlife managers are interested in protecting, e.g., wild forest reindeer (*Rangifer tarandus fennicus*) [6] and damage caused to crops and beehives by bears [7].

This paper considers conflict mitigation methods used to eliminate or deter large terrestrial predators (e.g., grey wolf (*Canis lupus*), brown bear (*Ursus arctos*), Eurasian lynx (*Lynx lynx*) and wolverine (*Gulo gulo*)) alongside those used to deter or eliminate marine predators (grey seal (*Halichoerus grypus*), harbor or common seal (*Phoca vitulina*), ringed seal (*Pusa hispida),* and Mediterranean monk seal (*Monachus monachus*)) in Europe. The potential impacts on animal welfare of these control methods are considered.

In some situations, lethal control is used to manage conflict. In Europe, Annex VI of the Habitats Directive and Appendix IV of the Bern Convention prohibit the use of certain means and methods of killing mammals including, for example, non-selective traps, and poisons [8,9]. Some countries have taken out reservations to parts of Appendix IV meaning that wolves can be killed or captured with snares in Belarus and Spain, and with traps in Latvia, Spain, and Ukraine [10]. Seals are also killed using traps or nets in some places in Europe though not usually as a conflict management tool but, rather, as a hunting method [3]. As shooting is the most commonly used method of lethal control for the predators covered here, this paper will focus only on the welfare impacts of shooting and not on other lethal control methods (see Section 3).

Some authors have recently started to evaluate the effectiveness of non-lethal methods used to manage predators, e.g., [11,12,13,14]. Khorozyan and Waltert, for example, assessed the effectiveness of various husbandry-related methods (fences and other enclosures, guarding animals, and herding), aversion methods (including acoustical, chemical, physical, and visual deterrents or a combination of these), and management methods (livestock breed replacement, calving control and supplemental feeding of predators) [14]. They concluded that the most effective interventions were electric fences, guarding animals, physical deterrents (protective collars and shocking devices) and calving control (i.e., shortening the calving season and limiting calf access to open grazing areas). 

However, decisions about how predators are managed are rarely based on non-biased scientific studies [13]. Other factors such as whether the mitigation method is ethically acceptable, the feasibility of applying the method, perceptions about whether the method will work or not, cost-effectiveness, target specificity and human safety might also influence which method is chosen [13,15]. This means that the most effective method, in terms of mitigating conflict, is not necessarily implemented and resources are sometimes wasted on inefficient or even counterproductive methods [13]. Lethal methods are sometimes applied when non-lethal methods would be more appropriate and/or effective. A lot of time and money is spent on protecting both livestock and carnivores and this is one reason why the lack of scientific evidence for the effectiveness of mitigation methods is of concern [13].

The welfare consequences of predator controls are also rarely taken into account. In a global assessment of bear management plans it was found that welfare was largely ignored in human–wildlife conflict management planning [16]. The authors acknowledged that measuring the welfare outcomes of conflict was not simple but, equally, that it is necessary. Indeed, welfare assessment models require detailed information about specific welfare impacts which are often difficult to measure or which are unavailable for wild animals.

In this paper, some potential ways for assessing how management methods impact on animal welfare are discussed. The most common method of lethal control (shooting) and non-lethal control methods used in terrestrial and marine environments are described and considered in terms of how they might impact animal welfare. It is not in the scope of this paper to carry out an actual assessment but, rather, to provide an introduction to the topic. 

## 2. Assessing the Welfare Impacts of a Control Method 

### 2.1. What is Animal Welfare?

The World Organization for Animal Health (OIE) defines animal welfare as “the physical and mental state of an animal in relation to the conditions in which it lives and dies” [17]. An animal with good welfare is “healthy, comfortable, well nourished, safe, is not suffering from unpleasant states such as pain, fear, and distress, and is able to express behaviors that are important for its physical and mental state.” In free-ranging wild animals, some of these criteria may be difficult to measure and, indeed, such a definition may not be applicable as some of the positive states may be unobtainable much of the time for wild animals and “unpleasant” states may be relatively common. Nevertheless, it is important to be able to assess whether specific human actions have an impact on animal welfare. How does a particular method of controlling predators or mitigating conflict negatively affect an individual wild animal’s welfare? Does the method reduce the opportunities for the predator to experience positive states or increase the possibility of it experiencing a negative state? How can this be determined?

Different authors have proposed different ways of considering animal welfare. Broom saw the welfare of an animal as its state relating to its ability to cope with its environment [18]. Therefore, considering what an animal has to do to cope, and how well or badly it copes can help us to understand its welfare state. Dawkins’ approach to welfare highlighted the importance of an animal’s needs and wants being met for it to be in a state of good welfare [19]. However she also pointed out that not all of its wants and needs will be met perfectly all the time, particularly in the wild, where animals are often in a state of compromise to survive in their environment. See Section 2.3 for further discussion regarding how these approaches may be applied to predator control.

When discussing specific welfare issues for a control method, the term humane is often used. The Oxford English Dictionary defines “humane” as “having or showing compassion or benevolence” and also “inflicting the minimum of pain,” [20]. When talking about animals, the word humane is generally taken to mean that the minimum amount of pain and suffering has been caused, particularly regarding killing methods [15]. It can, however, also be applied to non-lethal management methods by considering what impacts the method has on an individual’s welfare in terms of stress, disturbance and other negative consequences. The “humaneness” of a control method, therefore, refers to the overall impact that it has on the individual animal’s welfare. 

### 2.2. Considering Lethal Controls

To assess how killing methods affect welfare it is necessary to determine how severe a state of poor welfare is experienced before death and the duration of this poor welfare. In terms of negative welfare before unconsciousness or death, the animal may experience some or all of the following states: Breathlessness, thirst, pain, nausea, hunger, sickness, fear, anxiety, weakness, debility, helplessness, and other forms of distress [21]. How long the animal experiences the negative state(s) before it becomes unconscious or before it dies, can be used as a way to determine how welfare has been compromised. 

Littin et al. recommended the use of guidance published by the European Food Safety Authority (EFSA) for assessing pain, distress, and level of consciousness, which are all key elements in the assessment of the humaneness of a lethal method [22]. In 2018, EFSA published new guidance on the assessment criteria for stunning methods [23]. Though this guidance is focused on farm animals at the time of slaughter, it could provide some direction for assessing lethal control methods for wild animals. EFSA states that animal-based measures should be used to assess the onset of unconsciousness and death and the magnitude of pain, distress, and suffering before loss of consciousness. These measures can be neurological (such as electroencephalogram records), physiological (for example heart rate variability), behavioral (e.g., escape attempts) or physical reflexes (e.g., tonic-clonic seizures). 

Time To Death (TTD) has regularly been used as a way to evaluate aspects of the humaneness of lethal control methods and has been applied to various types of kills, including those related to whaling, badger culling, and beaver hunting [24,25,26]. However, Time to Insensibility or Time to Irreversible Unconsciousness (TIU) is increasingly seen as more appropriate than TTD, with many authors contending that suffering and/or negative impacts can only be experienced when animals are conscious. This contention also underpins the metrics and frameworks now being used for scientific evaluation of welfare [15,22,27]. TIU has been used, for example, to assess the humaneness of killing traps and livestock slaughter methods [28,29]. 

Methods used to kill animals should not cause avoidable pain, distress or other forms of suffering [30] and the killing “must be done in a way that causes minimum pain and reduces the time to death wherever possible” [15]. Humane killing, according to Broom, refers to situations where “the welfare of the animal is not poor just prior to the initiation of the killing procedure and the procedure itself results in insensibility to pain and distress within a few seconds” [31]. 

### 2.3. Considering Non-Lethal Controls

Although there is a general lack of data on the welfare impacts of wildlife management methods, this appears to be particularly true when it comes to non-lethal controls. One way in which they could be assessed is by considering how an animal’s ability to cope is affected (as proposed by Broom [18]). The use of some kind of deterrent to prevent carnivores from attacking livestock may, for example, have an impact on how the carnivore copes with its environment. It may be displaced from a habitat where it is accustomed to hunting and, for example, where it “knows” the terrain and where its prey may be found. Displacement could cause stress or hunger as the animal adapts to its changed circumstances and, thus, have an impact on its welfare. Similarly, taking livestock might have been relatively easy for the carnivore and securing wild prey may be more of a challenge requiring the expenditure of more energy to search for food. Depending on the type of deterrent used, the predator might experience a physical effect, for example a shock from an electric fence or discomfort from an acoustic deterrent which could prevent them, even if only in the short term, from coping with their environment. If the predator is quickly able to recover from the negative consequences of the control method, perhaps welfare will only be affected temporarily or mildly and the individual will quickly start effectively coping again without significant welfare consequences. 

If welfare is considered in terms of an animal’s needs and wants being met, as proposed by Dawkins [19], does preventing it from accessing a resource compromise its welfare? For example if a bear is prevented from accessing a beehive (something it wants) and it is reliant on that food source, then welfare could be negatively impacted. However, if there are other food sources available, then the bear may still be able to satisfy its wants and needs and be in a good state of welfare. Perhaps the animal will only experience negative welfare if its need for food is not satisfied over the long-term. After all, in the short-term, wild animals are often in a state of hunger, and animals are unlikely to be free from all negative states or experiences even for short periods [32].

### 2.4. Scientific Assessment Models

Rather than just assuming that the welfare of an animal will be affected in a specific way by a particular control method, a scientifically sound way of assessing and comparing the welfare impact of each method is required so that this can be taken into consideration when choosing the most appropriate method for a particular situation [15]. The Farm Animal Welfare Council developed the Five Freedoms (Freedom from Hunger and Thirst, Freedom from Discomfort, Freedom from Pain, Injury or Disease, Freedom to Express Normal Behavior and Freedom from Fear and Distress) for determining the welfare state of farm animals and they have long offered a starting point for welfare assessments [33]. They have been criticized, however, for giving the impression that it is possible to fully achieve these states when, in reality, they are “ideal” states and an animal cannot be kept completely free from all negative states during its entire life [32].

Elements of the Five Freedoms may be useful when assessing the welfare of wild animals in certain circumstances although they would need to be modified to be fully appropriate for assessing predator control methods. Such a modification may involve ranking the Freedoms in terms of importance; Freedom from Pain and Injury might be considered more important than Freedom from Hunger, for example, especially when the control method has been put in place to prevent the predator from predating on livestock and may, by design, lead to the animal going hungry.

To help address the potential limitations of the Five Freedoms, the Five Domains Model, developed by Mellor and Reid, identified areas where animal welfare can be compromised [34]. It has been updated since its original conception and now includes positive attributes, thereby showing how welfare can be enhanced (rather than only how it may be compromised), as well as giving more detail for negative affects which were originally included under the more general term “distress” [32]. The first four domains in the model are mainly physical/functional and cover “nutrition”, “environment”, “health”, and “behavior”. These four domains include welfare indicators which can be observed and/or measured using, mainly, animal-based indicators and, occasionally, resource/management-based indicators. The type, intensity and duration of those physical states can then be used to infer the animal’s “mental” state (or affective experience) as well as the type, intensity and duration of this mental state. This mental state is the fifth domain and it represents the accumulated impact of all the domains on overall welfare. The importance of the fifth domain when assessing animal welfare is widely recognized by animal welfare scientists [35]. The International Whaling Commission has started to consider how the Five Domains could be used to assess cetacean welfare [36,37] and a similar process for seals and terrestrial predators could be undertaken, considering their specific biology and needs. Beausoleil and Mellor provide a useful discussion of the benefits and limitations of the Five Domains for assessing welfare impacts of pest control methods [38].

Sharp and Saunders used the Five Domains to assess the humaneness of both lethal and non-lethal pest control methods [15]. Their assessment is a two-part process. Part A of their assessment uses the Five Domains approach to look at the impact of a lethal or non-lethal control method on overall welfare and the duration of the impact [15,34]. By combining the result of the overall impact on welfare (extreme, severe, moderate, mild, or no impact) with the duration of impact (immediate to seconds, minutes, hours, days, or weeks) in a scoring matrix, a humaneness score is obtained from 1–8 (where 1 is the most humane and 8 is the least humane) [15]. Part B of the assessment uses an approach recommended by Broom and evaluates the intensity and duration of suffering caused by lethal control methods [15,31]. The level of suffering experienced after the killing method has been applied but before the animal becomes insensible is determined (no suffering, mild suffering, moderate suffering, severe suffering, or extreme suffering) alongside the time to insensibility (immediate to seconds, minutes, hours, days, weeks) [15]. By combining these in a scoring matrix, a score from A-H is obtained (A is the most humane and H is the least humane). Lethal methods are assessed using Part A and B so that impacts on welfare before killing are considered as well as the actual method of killing. The humaneness score for lethal methods is, therefore, the result of combining the numerical score from Part A and the alphabetical score from Part B. The most humane method would score 1A and the least humane method would obtain a score of 8H.

When using this assessment, the authors recommended that assessment of humaneness needs to be considered alongside other factors such as target specificity, efficacy, practicality, cost-effectiveness, human health, and safety. The authors point out that their assessment model helps bring diverse stakeholders to a consensus regarding how a control method affects animal welfare. When carrying out an assessment it has to be assumed that “best practice” is being applied but that the animal should be given the “benefit of the doubt” i.e., if it is unclear whether an animal will suffer significantly it should be assumed that it will. If a lethal control method is not successful on its first application and has to be repeated then the animal will experience more intense overall stress. See [15] for how to apply Sharp and Saunders’ humaneness assessment method. It has been used to assess a variety of methods used to control terrestrial feral animals (donkeys, cats, camels, goats, horses and pigs) and pest species (foxes, birds, crows, moles, rabbits, rodents, wild deer, wild dogs, and possums) [15,39,40]. Its suitability for assessing control methods used for bears, wolves, lynxes, wolverines, and seals has not been tested, but as it has successfully been applied to a number of wildlife management scenarios, it may well be an appropriate model.

The Scientific Panel for Animal Health and Welfare of EFSA developed a qualitative Risk Assessment for evaluating the animal welfare aspects of the methods used for killing and skinning seals [30]. A risk assessment is different to a welfare assessment. In this case, the expert panel identified the hazards (i.e., events or influencing factors which could produce harm or have an adverse effect) and then characterized them in terms of intensity (negligible, mild, moderate, severe) and duration. Two different scoring categories were used for duration (one for netting and one for physical methods and firearms). The intensity and duration scores were then combined in a scoring matrix to determine the magnitude of an adverse welfare effect (magnitude could be negligible, minor, moderate or major). Some of the methods they assessed are hunting rather than management methods e.g., netting and physical methods (hakapik, slagkrok, club) whilst shooting with firearms is a method used in both hunting and conflict mitigation scenarios. The frequency of exposure to the hazard in the animal population was assessed and then the risk or probability of an adverse effect being experienced after the exposure to the hazard was determined and categorized as very likely, likely, unlikely or very unlikely. 

The Risk Assessment developed by EFSA was largely focused on seal hunts where the seals are killed, collected, and skinned for commercial or private use [30]. The Risk Assessment highlighted the importance of monitoring individual seals after a stunning or killing method has been applied to ensure that the animal is unconscious or dead before bleeding out takes place. Bleeding out ensures that the seal is dead before skinning commences. In the context of seal killing in conflict scenarios the majority of seals which are killed to prevent interactions with fisheries and/or fish farms are not subsequently bled out or skinned and, often, the monitoring element of the killing process is also missing, meaning that there is potential for seal welfare to be compromised. 

The EFSA Risk Assessment could, potentially, be adapted to assess the killing of terrestrial predators. However it may only be appropriate for assessing lethal control methods and not non-lethal methods.

## 3. Welfare Impacts of a Lethal Control Method: Shooting

### 3.1. Introduction to Shooting

Shooting is the most common method used for killing both terrestrial and marine predators. In some cases, animals are shot with firearms in conflict situations e.g., seal shooting in Scotland under the Marine (Scotland) Act 2010 to prevent serious damage to fisheries or fish farms [41]. In other circumstances, predators are killed as part of a regulated hunt and the carcass may be collected for use (e.g., bear hunting in Sweden) [42]. Illegal hunting of carnivores also takes place and is reported by various authors [43,44,45,46,47,48]. In some cases it accounts for a considerable percentage of carnivore mortality. One multi-year study reported that out of 94 monitored adult wolverines in Laponia (northern Sweden), of the 25 that were confirmed to have died during the study period (1993–2008), 36% (n = 9) were illegally killed [49]. 

When an animal is hit by a bullet, the cause of death will depend on where it is struck [50]. If the bullet hits major blood vessels or the heart, then fatal hemorrhage will occur and the animal will quickly become insensible [31,50]. If a bullet strikes vital parts of the brain then the animal will lose consciousness instantly and will die from heart and respiratory arrest and hypoxia [50]. A strike high in the neck (causing severe damage to the spinal cord) may cause the animal to become insensible immediately [31], although there is a risk that such a strike may only cause paralysis meaning that the animal remains conscious until death which could take several minutes [50]. Other spinal cord impacts could incapacitate the animal but may not be fatal unless large blood vessels are also traumatized. So, the animal could be sensible for several minutes before death. Most hunted terrestrial animals die from exsanguination because hunters target the thoracic area. Time from bullet impact to incapacitation (which is when the wounded animal is lying immobile on the ground and appears to be unconscious) due to blood loss depends on the rate of hemorrhage [50].

According to Stokke et al. the lungs are the best area to target when shooting a terrestrial animal because they are the largest vital zone that leads to rapid incapacitation and death because of massive hemorrhaging [50]. The heart is another preferred vital zone but it is a smaller target. When shooting a seal, the head is the recommended target in most cases, although the upper neck just behind the head is acceptable in some cases/countries [30,51].

If an animal is shot so that it is immediately rendered insensible, then there is no welfare issue for this individual animal and its death can be considered humane [18]. The potential for negative welfare impacts arises in cases where the animal is not shot properly or where other animals (such as dependent young) are affected. In commercial seal hunts a key part of ensuring that seal welfare is not negatively impacted after the animal is shot, is the subsequent checking and bleeding out of the seal [30]. An Independent Veterinarians’ Working Group on the Canadian Harp Seal Hunt recommended that checking should be carried out by palpating the skull to ensure that the brain is destroyed rather than checking for the absence of a blink reflex [52]. As seals in commercial or subsistence hunts are often shot on land or ice to aid recovery of the carcass, such checking is relatively easy to do [3]. The checking of the state of consciousness of a terrestrial carnivore after it has been shot should also be possible and, in cases where lethal control is undertaken by hunters who wish to recover the carcass, checking may be routinely undertaken. When seals are shot for management purposes, however, they are often shot whilst in the water and so the subsequent checking of the carcass may be impossible despite this being recommended in some codes of practice, for example in Scotland [51]. Recommendations to commercial seal hunts advise against the shooting of seals in water, in part, because of the inability to confirm irreversible unconsciousness [52].

Variables that can influence the success of a shot include type of weapon and ammunition (including its velocity and mass), range, skill of the shooter, movement and direction of the animal, exposure time, weather, terrain and the attitude and position of the marksman including whether or not they are in a moving vehicle [53,54]. To reduce the chances of wounding an animal, marksmen should not rush the shot nor shoot an obscured or moving animal and they should be in a comfortable position ideally using a gun rest [55]. Monthly shooting practice can help reduce the possibility of wounding an animal. Seals are more likely to be injured (rather than killed) when the animal is shot from too far away, from an unstable platform, with inappropriate firearms or ammunition and if the marksman makes a mistake [30].

### 3.2. Wounding

When a shot hits an animal with insufficient force and/or accuracy it may be wounded rather than killed [30]. Animals that are wounded may only die after hours, days or even weeks, with the period before death most likely resulting in poor or very poor welfare for the individual animal [31]. The seriousness of a wound when an animal is shot is not necessarily proportional to the suffering it experiences [53]. A seriously wounded animal may die within a few minutes or hours, for example, and a lightly wounded animal could take days or weeks to die or recover from the wound. Non-lethal wounding can lead to a number of welfare issues such as the disabling effects of the injury e.g., inability to feed, escape threatening situations and perform particular functions because of damage to a specific area of the body, infection leading to sickness, pain and discomfort and chronic psychological effects [56]. Using Broom’s definition of welfare, an animal with such wounding issues may be unable to cope with its environment and, depending on the severity of the injury, welfare could be severely impacted [31].

There have been relatively few scientific studies that have attempted to assess wounding rates in hunted populations of terrestrial mammals meaning that attempts to reduce wounding and suffering are made without the necessary information [50]. During a pilot badger cull in the UK, it was calculated that after a rifle shot during controlled shooting, between 6% and 19% of badgers are not recovered [25]. In some of these cases the badger may have been missed by the shot but, in others, it could have been hit and injured. For example, out of 88 observed shots, 10 (11.4%) did not result in a carcass being recovered. Six of these shots were reported as misses but in one case signs of badger injury were found at the site and in three cases behavior indicating a hit was observed. Aebischer, Wheatley and Rose found that out of 2179 deer that were hit by the first shot fired at them, 2026 (93%) were killed outright and 153 (7%) were wounded [55]. One hundred and twenty-five (81.7%) of the wounded deer were killed with a subsequent shot but 28 (18.3%) were lost or escaped wounded. It has been estimated that at least 9% of shot brown bears and 19% of shot lynx are wounded and not killed immediately [57]. 

Recognizing whether an animal has been wounded rather than killed in the field is important. Stokke et al. have developed a model to establish acceptable animal welfare outcomes for hunting systems for a range of body masses and terrestrial game species [50]. With a rough estimate of body mass of the shot mammal and a measurement of flight distance, hunters can consult the wounding thresholds that have been determined in order to estimate animal welfare outcomes. Therefore, if a shot animal’s flight distance is further than the indicated wounding threshold, the hunter can assume that the animal is wounded. They recommend that the model can be used to determine the minimum caliber requirements for species. It should be noted that other factors can affect the threshold distance such as the steepness of the terrain, density of vegetation, animal stress level and snow depth [50]. 

In the water, it is far harder to assess whether a shot seal has been wounded or whether it has been killed instantly. There have been cases of seals in Scotland not being killed by the first shot [41]. The EFSA Risk Assessment found that it is “very likely to likely” that shooting seals is an effective way to kill them and that suffering would be “negligible, especially if death was ensured by another method of killing” [30]. Though ineffective shots are “unlikely”, if the animal is wounded and remains conscious “then suffering will be high” [30]. A wounded seal may drown which is considered a painful and distressing way to die [58]. The EFSA expert panel deemed that if a seal is not killed by a first shot and re-stunning is necessary that it is “likely” that this will be effective and suffering will be “low”.

When killing seals in bad weather (e.g., poor visibility) and on bad habitat (e.g., open water), the chances of an effective hit are reduced and greater suffering is “likely” [30]. The authors of the EFSA report concluded that when seals are shot in locations where reaching the shot animal was difficult (e.g., in open deep water) then there was an unknown risk of causing unavoidable pain, distress and suffering [30]. As seals shot in management situations are rarely checked or the carcasses recovered then this may be a significant source of animal suffering in some fisheries/around some fish farms [41]. Nunny, Simmonds, and Butterworth report that there is no international standard for how seals should be shot and, therefore, it is possible that seals are being shot in circumstances that lead to suffering [3]. The Seal Management Code of Practice produced by the Scottish government states that “steps should be taken to ensure against a prolonged and painful death including locating and humanely dispatching injured animals” [51]. It is not specified how to locate a seal which is injured and subsequently dives or swims away. 

Whether or not the seal is correctly monitored after shooting is as important as the actual killing method used. EFSA considered that the probability of effective post-shooting monitoring taking place in seal hunts was “very unlikely” to “unlikely” and that the consequences for the seals could range from negligible to severe [30]. If this ineffective monitoring takes place in seal hunts when the sealer approaches the seal at close quarters to subsequently skin it, then it might also be considered unlikely that effective monitoring will take place in a management situation. The low number of seal carcasses that are collected by marksmen in Scotland suggests that in some places this is indeed the case [41].

### 3.3. Impacts on Non-Target Animals

As well as assessing the welfare impact of shooting an animal in terms of how that individual is killed, the potential impact on other animals should also be taken into consideration. This is of particular importance when the shooting of a female leaves her dependent young orphaned and unable to fend for themselves, as they will starve to death. Close seasons can help protect dependent young. In Finland and Sweden female bears with cubs and cubs younger than one year are protected [59,60]. Not all countries in Europe have close seasons for seals during the breeding seasons and, therefore, lactating mothers with dependent pups could be targeted in some places e.g., Scotland [3]. 

The removal of individuals can also negatively affect social structures and population dynamics [61]. The hunting of male bears, for example, led to a decrease in cub survival during the mating season because of sexually selected infanticide. The distribution of male bear removals through hunting could be a more important factor for social structure than the overall number of males that are killed. Animal behavior can also be affected by the mere presence of hunters as animals try to avoid them. This can have a negative impact on welfare if activity and feeding patterns are affected e.g., bears in Sweden changed their daily behavior during the hunting season [62].

### 3.4. Welfare Impacts of Events Leading up to Application of Kill Method 

Sharp and Saunders’ humane assessment takes into account the period before the killing method is applied [15] and in some hunting scenarios this is important if the predator is being hunted in a way which may have a negative impact on its welfare. Hunters in Sweden, for example, sometimes use dogs (either on or off the leash) to pursue bears and to keep them in place for the hunter to shoot [42]. Hunting with dogs can cause more suffering than stalking [56]. A study comparing hunting with dogs to stalking found that the hunted animals experienced severe physiological effects reflected by high plasma concentrations of cortisol which is associated with extreme stress [63]. The pursued animal may experience exertion, fatigue, respiratory distress, exhaustion, fear and injuries during the chase, including bites from the hunting dogs [56]. 

## 4. Welfare Impacts of Non-Lethal Control Methods

There are many non-lethal methods for reducing conflict with predators including enclosures, deterrents, the guarding of livestock and a variety of other management methods. Using a combination of methods may be the most effective way of protecting livestock [64]. The effectiveness of conflict mitigation methods depends on the context in which they are implemented and whether the specific problem is targeted [12]. In this section, an introductory exploration of the literature relating to non-lethal control methods and their potential welfare impacts is made. A welfare assessment is not specifically attempted but, rather, some ideas about how welfare might be impacted are presented based on the available literature and welfare assessments of other species/other scenarios.

### 4.1. Enclosures

In both terrestrial and marine environments, enclosures are used to protect human interests including livestock, crops, and farmed fish. On land, the type of enclosure or barrier used to protect livestock, including its height and material, will depend on the husbandry system in which the animals are being raised. Permanent fencing might be appropriate in smaller operations where night corrals or small pastures are used, for example [65]. Fences need to be high enough so that predators cannot jump over them and without any gaps. Properly constructed and maintained carnivore-proof electric fencing such as 5–7 strands of high tensile wire with very high voltage can work effectively in permanent pastures against many species of carnivore including wolves and bears [66]. In some husbandry systems, the use of enclosures may not be possible e.g., in Norway, Sweden, Finland, and Russia semi-domestic reindeer (*Rangifer tarandus*) are free-ranging all year round meaning they are exposed to wolves, brown bears, lynx, and wolverines [66]. The main method of protecting them is via lethal control of predators.

Portable fencing made from multiple electric fencing strand, wire netting or mesh and portable panels may be suitable when permanent fencing is not appropriate [65]. It can be used in open pastures and can be useful in emergency situations or during periods of increased risk, though it is not as long-lasting or as strong as the fencing for permanent pastures [66,67]. Solar-powered electric fences work well at protecting beehives from bears [68].

Fladry is another type of barrier used to deter carnivores from gaining access to livestock. Fladry is the use of flags hanging from ropes or attached to fences to create a barrier to deter predators from attacking livestock [11,69]. It can be hung from an existing fence or placed outside of the existing livestock fencing as a preliminary barrier [65,70]. Electrified fladry (also known as turbo-fladry) combines the use of flags and an electrified barrier [71,72]. When traditional fladry fails because wolves start to get habituated to it, electrified fladry offers an additional deterrent because the wolves receive an aversive shock if they come into contact with it. The use of non-electrical fencing (and, potentially, fladry) to exclude animals from a food source could be considered to have a mild impact on their welfare as found by Baker et al. in their assessment of rabbits [39]. In that case the impact was found to last for days and that once the animals had adapted their behavior to the presence of the exclusion method, there was no further welfare impact [39]. Electric fences, on the other hand, can cause aversive sensations and pain [73]. (See Section 4.3 for welfare impacts of aversive stimuli).

In the marine environment, anti-predator nets at fish farms work by surrounding either the whole cage system or individual cages from the surface of the water to the seabed and can be of a curtain, skirt, or box design [74]. Some types of netting material have been found to be more effective at preventing seals from attacking fish and new materials, which combine polyethylene with steel or copper cores, are said to be “predator resistant” [75]. High Density Polyethylene (HDPE) is less extensible than nylon and may help prevent predation [76]. As with other exclusion methods for wildlife, the welfare of the seals is unlikely to be negatively impacted if there is sufficient wild prey available for them to feed on and if the exclusion method is implemented before the seals have begun to rely on the fish farm as a food source. 

### 4.2. Guarding

The role of shepherds in guarding their flocks from predators goes back into pre-history and it is a method which is still used in many areas, often in combination with livestock-guarding dogs (LGDs) [66]. LGDs are also used alongside fencing and they may prevent carnivores from entering enclosures and can mean the presence of a shepherd is not necessary [77]. A combination of several LGDs and night enclosures was found to prevent the majority (>95%) of livestock kills by wolves in the French Alps [78]. LGDs are also used in husbandry systems without enclosures and are considered essential for the protection of flocks in areas with a high risk of predation and for allowing grazing in shrubby areas or where flocks travel long distances each day to find grazing and where it would otherwise be difficult to protect them [77]. Herds should be protected by at least three dogs particularly if they are at risk from wolf attack because the wolf pack may divide the livestock and a single dog would not be able to protect the entire herd [64]. Groups of dogs can work together to divide the work of protecting the pasture and staying with the herd. There is no guarantee that there will be no predation or damage caused by large predators when LGDs are present and certain terrains or weather conditions may give predators the advantage and stop LGDs from warning of an attack [77].

Wolves rarely fight with LGDs, so injury of either animal is unlikely [64]. However, there have been occasional cases of LGDs being killed by wolves [77] and the welfare of LGDs is something to bear in mind when considering the overall animal welfare consequences of this predator control method. LGDs may also kill medium-sized carnivores or wild prey species in some circumstances and so it may not always be appropriate to consider them a “non-lethal” control method [79].

There is no existing equivalent of LGDs and shepherds for use in the marine environment to protect fish at aquaculture sites, although a large model orca (*Orcinus orca*) emitting orca vocalizations has recently been deployed in Scotland to try to deter seals [80]. The potential impact of this on seals is unclear but see Section 4.3 below for how it could affect them. 

### 4.3. Aversion

Deterrents, repellents, and aversive conditioning (including the use of chemicals, sounds, and light) have all been proposed as methods to help instill fear in wolves to prevent them from coming into close contact with human settlements and livestock [81,82]. 

Lacing carcasses with something aversive has been used as a method to attempt to deter carnivores from attacking livestock [66]. Various trials have looked at using lithium chloride with both terrestrial predators such as coyotes (*Canis latrans*) and marine predators such as California sea lions (*Zalophus californianus*) [83,84]. In Tasmania, Australian fur seals (*Arctocephalus pusillus doriferus*) were seen convulsing, vomiting, and leaving the area after eating bait laced with lithium chloride at a fish farm [85]. This would have been a negative impact on the seals’ welfare, even though it may only have affected them in the short-term. The use of such aversive techniques does not necessarily cause a long-term change in behavior. California sea lions that were given lithium chloride as a conditioned taste aversion agent to prevent them taking steelhead trout *(Oncorhynchus mykiss*)*,* did not seem to associate the conditioned taste aversion with the act of foraging and continued to forage after vomiting [83]. This has also been an issue when using lithium chloride to prevent coyotes from killing sheep. Coyotes do not necessarily associate the aversive reaction experienced after eating a treated bait (in mutton or on a carcass) with a live sheep or lamb [84]. The use of netting impregnated with a strongly disagreeable taste has been proposed as a possible future work area for those attempting to deter pinniped predators at fish farms [74]. 

Lights and sirens, blank handguns, Movement-Activated Guard (MAG), and Radio-Activated Guard (RAG) devices have been used to scare wolves [72,86,87]. Both MAG and RAG devices emit strobe light and a variety of sound effects (including gunfire, helicopter sounds and shouting) when activated. The first is triggered by the movement of a large animal close to the detector whereas the second relies on the predator being fitted with a radio-collar which triggers the lights and sounds when the animal approaches the area being protected. Such devices may elicit fear in the predators which could be a negative welfare impact depending on duration and subsequent effects on behavior such as interfering with feeding and use of space [88,89]. The use of some aversive methods can also have an impact on the welfare of the livestock or farmed fish which are being protected from predators [90,91] and this needs to be taken into consideration when choosing an aversive control method. 

Electric training collars have also been used on wolves [86]. These collars have caused domestic dogs to exhibit stress and pain in some circumstances [89] and it is possible that wolves could experience similar outcomes. However, some studies have found that results with wolves are highly variable [86]. Some wolves found the stimuli from the collar very noxious and reacted by jumping, yelping and running away whereas other individuals hardly reacted and continued to carry out the behavior which had prompted the collar to shock them. 

The use of any type of collar first requires the capture of the wolf and this procedure can have negative welfare impacts. If an anesthetic is being used, direct effects of the drug itself (e.g., respiratory depression, shock, vomiting, and hyperthermia) can have a negative impact on welfare [92]. There is also the risk of indirect effects, such as drowning during induction (if the animal is immobilized near water), pneumothorax caused by a misplaced immobilizing dart or trauma from the dart impact [92]. Secondary effects may cause negative welfare as well, such as trauma from the capture method, long-term effects from chasing or stress, separation of mother and offspring, and subsequent problems with the collars [92]. Direct, indirect, and secondary effects can all lead to the death of the animal involved although, if correct protocol is followed and experienced capture teams carry out the procedure, mortality and negative welfare impacts can be kept to a minimum [92]. 

Aversive methods using electricity have also been used to deter seals. Electric field barriers are effective at deterring seals in some circumstances [93]. In freshwater, harbor seals avoided the sections of a gill net with an electric deterrent system [94]. Grey and harbor seals can detect low voltage pulsed electric fields and the response elicited varies according to combination of pulse length, amplitude and repetition rate [95]. They can be prevented from entering a small area using a low voltage, short duration pulsed electric field. Although seals demonstrated an avoidance response, they did not seem agitated or injured by the exposure to the electric field, though this could be a possible outcome and could, therefore, have a negative impact on welfare [94].

At fish farms acoustic “seal scarers” or “pingers” can be used to deter seals. Acoustic Deterrent Devices (ADDs) alert seals that there are nets in place whilst Acoustic Harassment Devices (AHDs) are louder and aim to keep animals away from the cages by making sounds which frighten the seals or cause them discomfort [96,97]. Both ADDs and AHDs might, potentially, have negative impacts on target species such as causing damage to the seal’s ear and masking the sounds which they use for communication, orientation or prey detection thereby causing the seals to avoid part of their habitat and increasing stress hormone levels [98,99,100]. Non-target species such as cetaceans can also experience negative welfare [98,99].

Recordings of orca vocalizations have been used to scare harbor and grey seals away from aquaculture sites and at one site in Scotland the emitted sounds come from a fiberglass model orca [80,97]. The effectiveness of such methods has yet to be evaluated. Their impact on seal welfare is also unclear, although Cape fur seals (*Arctocephalus pusillus pusillus*) at risk of predation experienced a stress response [101] and it is possible that the use of predator vocalizations could elicit a similar response.

Any method which aims at scaring or causing an aversive reaction actually aims at eliciting the negative responses covered by domain five (anxiety/ fear/ pain/ distress) in order to prevent the predator from attacking the farmed fish or livestock. 

### 4.4. Promotion of Wild Prey and Supplementary Feeding

Methods to deter predators from attacking livestock are more effective when wild prey is also promoted and the restoration of habitats and ungulate populations are recommended by some authors [66,81]. In Belarus, wolves took more domestic animals, mainly cattle, during years when wild ungulate populations were at their lowest from poaching and uncontrolled exploitation [102]. In Slovakia, the relatively high availability of alternative food has meant that damage per carnivore is lower than in other areas [103] whilst, in central Portugal, the Iberian wolf’s diet is largely made up of livestock because of the low diversity and density of wild prey [104]. In Fennoscandia, lynx and wolverines are largely dependent on semi-domestic reindeer [66]. This issue could also be relevant in marine environments. Conflict between monk seals and fish farmers in Turkey, for example, was considered greater because wild fish stocks had been reduced by overfishing, and hunger was compelling the seals to attack the fish farms [105]. 

One approach used to divert bears away from human settlements, crops, and livestock is to artificially feed them and this practice has been used in many countries including Bosnia and Herzegovina, Croatia, Romania, Serbia, Slovakia, and Slovenia [106,107]. In other countries, e.g., Sweden, such supplementary feeding is discouraged because it is considered to increase conflict [107] and some authors have recommended that artificial feeding should be avoided [7]. From a welfare point of view, supplementary (or diversionary) feeding could lead to improved physical condition for predators that have been struggling to find enough food [108]. Potentially negative impacts include increased potential for disease transmission, disruption of animal movement patterns and distribution which could lead to conflict between individuals.

### 4.5. Habitat Use and Management Factors

The way livestock are managed can have an effect on how likely they are to be attacked by predators. As carnivores may rely on cover when making their final approach before attacking, depredation can be mitigated by keeping livestock in open areas and focusing mitigation efforts on areas close to forests [66,109]. More specifically, herds that graze in areas favored by individual carnivores are more likely to be attacked because of the increased likelihood of the carnivore coming into contact with the herd and because it feels secure enough to make an attack [66]. Herds which have been identified as being at risk can be prioritized when protection measures are being put in place. The risk of livestock being attacked by predators also increases when more livestock are present, when sick or pregnant animals stray and when humans are far away [82]. In Norway, sheep that are brought down from summer pastures earlier in the year are less exposed to predators than those which stay in pastures until later in the year [110]. Similarly, beehives close to forest areas are more likely to be attacked by bears than those further away [111]. 

Conflict in the marine environment can also be location dependent. Some authors have found that fish farms that are situated close to seal haul-out sites are more likely to be attacked by seals [85] and it has been recommended that fish farms be located away from core seal habitat [3]. It is noted, however, that in some places, such as Scotland, seals are present throughout coastal areas and most fish farms are located within 3 km of the nearest seal haul-out site [74]. In Irish fisheries, interactions with seals can be reduced by relocating fishing effort after seal depredation or avoiding fishing near haul-out sites [5].

If humans adapt their habitat usage to avoid conflict, there is not necessarily any negative impact on predator welfare if predators still have access to appropriate habitat and wild prey [43]. However, if the way that land is used leads to habitat fragmentation and wildlife populations being confined to inhospitable areas, welfare may be negatively impacted as the predators may be exposed to higher risks of mortality as they seek appropriate habitat and prey or if they have to use more energy to forage efficiently [112]. 

Traditional farming and fishing techniques can sometimes be modified to reduce conflict with carnivores. For example, beehives can be protected from attacks by bears if they are placed on platforms at least 3 m above the ground and if they are also protected by electric fencing [113]. Where wild fish are at risk of predation by seals whilst in the fishing net, modifications to nets can help reduce predation. In a salmon fishery in Scotland modifications to the size of the net entrance helped increase the number of salmon landed and reduced fish hesitation in the outer part of the net – something that is considered important for reducing depredation [114]. Modifications made to trap nets in the Baltic Sea showed that using strong seal-safe netting and different designs such as the pontoon trap can reduce the amount of damage that seals cause to caught salmon [115,116]. This method of conflict mitigation should be non-lethal but, depending on the type of net or trap used, there is also potential for seals to drown if they get stuck inside the apparatus [115]. Because of their diving adaptations, seals that are trapped underwater do not lose consciousness quickly and the process leading to death can, potentially, last tens of minutes meaning that the potential for trapped seals to experience stress, pain and suffering is prolonged [30].

Possible ecological reasons for human–wildlife conflict need to be examined. If conflict is driven, for example, by reduced food supply but wildlife managers choose to lethally control or hunt predators because they think the population density has increased, they may end up putting more pressure on an already struggling population [117]. A study in Canada found that more grizzly bears (*Ursos arctos horribilis*) were killed due to conflict with humans when there was a decrease in food availability (in that case salmon) [117]. The vast majority of attacks on humans (81%) and 82% of conflict kills of grizzly bears took place when the bears were in hyperphagia (a period of intense calorific demand). By taking such seasonal changes into account, livestock holders can specifically target mitigation methods. In Europe, bears in hyperphagia rely on getting enough calories from a berry-based diet which, ideally, requires foraging during the day when they can locate feeding sites with the highest berry densities [62]. When hunting is permitted during periods of hyperphagia (as is the case in some countries such as Sweden) bears may experience negative welfare impacts as hunting provokes changes in behavior including increased movement patterns, less time spent foraging during the day and loss of nocturnal resting periods to compensate for this [62]. Metabolic rates can also increase under the threat of hunting which, for bears relying on berries for nutrition, can be another challenge. 

Livestock holders should keep good records of any incidence of conflict with predators as this can help identify trends such as specific times of year when livestock are most at risk or areas of vulnerability [65]. Management methods can then be specifically targeted at the problem. Both the behavior and biology of the species being managed needs to be fully understood and then non-lethal control methods can be effectively employed [118]. A study of sheep farms in Slovenia found that 78% of wolf attacks took place at night showing the importance of understanding wolf behavior and the necessity of protecting livestock at night [119]. 

The mitigation method applied may be dependent on the time of year. For example, during periods when wolf cubs are very young, it might be necessary to stop using LGDs and to use alternative methods so that the LGDs do not come into conflict with wolves (denning wolves see dogs as a threat and may seek them out and kill them) [65]. 

In Scotland, frequency and intensity of depredation at fish farms by seals depends, to a certain extent, on the growing or production cycle of the salmon [74]. Attacks are infrequent at the beginning of the production cycle and then increase slowly for the first seven months before plateauing for the remaining months. Intensity of attack is highest at around months 9 and 10 of the process, which is when the most fish per month are lost to seal predation. This coincides with the time when fish are moved from smolt-type netting (15 mm mesh) to grower-pens with a larger mesh (25 mm). Intensity of depredation then decreases perhaps because fish become more difficult to catch. The peak intensity of seal depredation takes place in December which is immediately after the grey seal breeding season during which adult animals fast.

How dead livestock are dealt with is important. Wolves can be attracted by the smell of carcasses or sick animals and, once drawn to an area, may then move to attack other members of the herd [65,82]. Carcasses should be burnt or buried rather than left to decay in the open [65]. In the marine environment, the removal of dead fish at fish farms is considered an essential task which must be carried out daily and the use of seal blinds to cover the dead fish basket is also recommended [120]. A seal blind is the area at the bottom of the net where a thicker square of netting material conceals dead fish from seals approaching the net from underneath [74]. Khorozyan and Waltert also found controls around calving to be important for reducing conflict [14]. Afterbirth can attract carnivores and so it is necessary to plan the timing and location of calving [65]. Management methods such as these do not appear to have a negative impact on predator welfare.

### 4.6. Translocation

Moving predators from an area of conflict to another location has been used as a method of conflict prevention in some circumstances. To successfully translocate terrestrial carnivores the animal has to be moved far enough away so that it cannot return to the area of potential conflict, it needs to be moved to a place with suitable habitat and where it is not going to come into conflict with other conspecifics [82]. However, translocation is often ineffective and appears to have negative impacts on welfare. Over a ten-year period in Tasmania, Australian fur seals were trapped at salmonid farms and translocated [121]. Of nearly 600 capture events, 52% involved seals that had been captured more than once. Some seals were easier to catch than others (some were declared not “trappable” by the authors) and it was clear that translocation did not prevent seals from interacting with fish farms. The Sea Mammal Research Unit at the University of St Andrews in Scotland has been developing a method to trap seals in rivers using a net system before translocating them but there is concern that if seals are translocated they could quickly return to the capture site [93]. Indeed, many terrestrial mammals which have been translocated have subsequently undertaken long journeys with some even returning to the point of capture [122]. 

If the translocation leads to aggression among conspecifics or behaviors such as infanticide due to social disruption, then there could be negative consequences for animal welfare [82]. Translocation can also cause acute and/or chronic stress which can have an impact on an individual’s health and cognitive abilities [123]. Stressed animals may struggle to find prey and may go hungry or waste time and energy seeking food which can lead to a decline in body condition. Not only may the translocated animal suffer stress, but animals in the source population may also be affected if established social relationships are disrupted by the removal of an individual [124]. The possibility of translocated animals carrying diseases or parasites into a new population needs to be considered, as well as the fact that translocation can cause stress-induced disease. In general, translocations are considered to have clear welfare costs for the individual animals and that they, often, do not achieve their aims [125]. 

### 4.7. Fertility Control

Fertility control or sterilization are methods which are rarely used to control large mammal predators because, in some circumstances, they may be contrary to conservation goals as well as being ethically questionable and expensive [12]. If used, fertility control may reduce livestock losses, for example, by eliminating the need for predators to provide food for their young [12]. Melengestrol acetate has been successfully used as a contraceptive for carnivores in zoos and immunocontraceptive vaccines have been effective in seals and bears [126]. However, fertility control does not only affect birth rates but can also affect the physiology and behavior of treated animals which could lead to welfare issues [31,126]. In some cases, physiological responses may lead to improved health and body condition [126] but, in canids, some contraceptive methods have been associated with higher incidence of uterine pathology [127]. 

If the animals need to be captured to be injected, to have an implant inserted or to be sterilized surgically there may be a risk of infection, multiple dose toxicity, or capture trauma [128,129]. If the animal has been immobilized, it may have negative welfare experiences during and immediately after recovery such as vomiting, hypothermia, and hyperthermia, and it may be at greater risk of attack from other animals or poachers [129]. Fertility control can have welfare advantages when compared to lethal control. It is less likely to cause social perturbation and disease transmission than culling which can prompt increases in both due to disruptions to social organization and increases in animal movements [126,130]. However, preventing animals from breeding and caring for their young may deprive them of an opportunity to experience natural reproductive and parental behaviors and, thus, a state of positive welfare [131].

## 5. Discussion

In both the marine environment and on land, predators are managed using various lethal and non-lethal controls, many of which may have a significant impact on their welfare. However, assessing welfare impacts and, in particular, comparing them between different controls, is only in its infancy. How conflict with predators is managed depends on a number of factors including the legal and protected status of the predator, whether or not it is considered a pest or invasive species, any management plans that are in place, the resources available and societal and cultural norms [132,133]. Despite the apparent high public and political interest in such matters, there is a lack of scientific evidence for the effectiveness of many control methods [12]. The use of lethal control (for example in the case of wolf management) does not always achieve the desired result of conflict reduction [134]. Lethal control can, for example, alter carnivore social structure and cause more individuals to migrate, take over and start depredating in an area, thus exacerbating a problem rather than solving it [12,135]. Predator controls that have not been proven to be effective should not be used and more studies need to be carried out to determine the best ways to approach different conflict scenarios [14]. 

It is essential that the animal welfare impacts of any control method should be rigorously considered and included in wildlife management planning. A welfare assessment needs to be scientifically-based, well documented, objective, repeatable, transparent, and open to review [30]. It is essential that any assessment of the risk to animal welfare should be done from the point of view of the animal. The Sharp and Saunders model is appropriate for assessing non-lethal controls as well as lethal controls [15]. Baker, Sharp and Macdonald and Beausoleil et al. provide examples of applications of this model as well as helpful reviews of its usefulness and potential challenges [39,40].

Once a welfare assessment has been carried out, any potentially negative welfare impacts can be balanced with the welfare benefits experienced by the livestock or fish which are affected by predator attacks. Similarly, by changing the behavior of predators there may be implications for the welfare of their new target prey if they switch from domestic to wild prey. This is a topic which deserves further consideration.

Wild animal welfare assessment may take into account how many animals are affected by a particular action, what the action affecting them is and how long it lasts and the capacity of the animal to experience suffering [136]. However, the *level* of suffering of each individual is as important as the number of animals affected by a welfare impact [39]. The ideal may be “the least animal welfare harms to the least number of animals” as recommended by Dubois et al. [132]. It is, therefore, important to consider which animals are involved in conflict scenarios. Indeed, many individual carnivores which have access to domestic animals or humans do not come into conflict with them [82] and some authors have recommended that only “problem” individuals should be targeted [12]. This could have some positive repercussions, such as more cautious animals, which avoid humans, passing on their genes or their learned behavior to future generations [82]. Königson et al. identified “problem” seals which specialized in raiding salmon traps and found that removing these seals meant that fishermen experienced a decrease in seal damage [137]. However, Artelle et al. found that removing “problem” grizzly bears did not reduce the frequency of conflict [117]. This highlights the importance of determining whether a particular management style (e.g., removing certain individual animals) is likely to be effective or not.

Moreover, it does not always follow that in predator control situations it is the “problem” animals that are removed [82] and during attempts to remove “problem” animals, non-target individuals are often killed instead [135]. In some situations, whatever the methods used to prevent predation, it may be impossible to stop certain individual predators if they are highly motivated and determined to reach a particular resource [138]. It should also be recognized that in many conflict situations there may not actually be “problem” individuals [88]. Baltic ringed seals, for example, were found to range widely between foraging sites and, therefore, removing one individual seal was considered unlikely to reduce conflict [139]. Indeed, it is not always easy to identify the exact problem—whether it is one individual animal, all individuals of one species, or whether more than one species of carnivore is a threat [12]. 

What stakeholders believe about the animals that they think are interfering with their interests and what is actually occurring may not be the same thing. So, for example, there may be situations where it is believed that there is a “problem” individual, when in reality there is not. It is important, therefore, that the results from any studies into the actual mechanisms of conflict are shared appropriately with those affected and that differing human values are taken into account when planning how to manage conflict [132].

The killing of specific individuals is not the only scenario in which lethal control is recommended. Hunting is sometimes promoted as a way of creating avoidance behaviors in carnivores [140]. For example, some stakeholders believe that by killing some wolves, other wolves are kept shy of humans and places that humans utilize and that hunting can, therefore, help to protect livestock by altering predator behavior [141]. Fishermen in Finland have, similarly, argued that shooting grey seals makes them “wild” so that they stay out at sea, keeping them away from fishing grounds [142]. It may be possible to use deterrents to elicit this fear and avoidance behavior without resorting to lethal control. However, as mentioned previously, the effectiveness of the deterrent needs to be proven so that efforts are not misdirected. 

As well as keeping carnivores wild and fearful of humans, it is also necessary for people to maintain the appropriate level of respect for wild predators. This may be particularly necessary in areas where large terrestrial carnivores have returned after a long absence [81]. If the necessary separation of humans and carnivores is achieved and there is sufficient wild prey, conflict can be minimized.

In this paper the focus has been on large carnivores and, in the marine environment, seals but it is noted that other species come into conflict with humans under similar circumstances. One species that may be of interest when considering future efforts to mitigate conflict is the Eurasian otter (*Lutra lutra*), an animal that bridges the gap between the terrestrial and aquatic worlds and is known to prey on fish at aquaculture sites and in angling rivers e.g., [143]. In the UK, for example, many fish farms have been established at a time when otter populations were low and, now they are recovering, conflicts may increase [144]. This, potentially, will require the use of increased anti-predator controls which could have implications for animal welfare if the controls are not properly assessed for their impact on welfare. 

## 6. Conclusions

The control of predators is a complex topic that requires more research and more data-sharing—including between those working on such issues on land and at sea. Whilst in some areas, such as the non-lethal methods applied, there is limited overlap between marine and terrestrial species, there are common issues on land and at sea that should be explored further. These include:

- the establishment of standards for shooting (highlighted as an issue for seals in Europe by Nunny, Simmonds and Butterworth [3]). Whilst these have to be species-specific to reflect anatomy, habitat and behavioral differences, there should be discussion about what is effective and what best safeguards animal welfare;
- greater consideration of ‘perceived’ versus ‘real’ negative impacts of predators and how to deal with the expectations of stakeholders—an issue that seems to affect all species in conflict with humans [132]; and
- greater effort to educate affected stakeholders in the appreciation of the complexity of predator controls and related welfare issues and to ensure that predator management plans include an animal welfare component.

This will help to ensure that effort goes only into truly effective measures and, when applied in combination with appropriate welfare assessments, will minimize negative animal welfare consequences for both predators and their prey. Further work is required to develop standardized and widely accepted methods for assessing wild animal welfare so that the lack of data regarding the welfare impacts of predator control methods for both terrestrial and marine mammal predators, and particularly for non-lethal methods, can be addressed. Assessment models that have already been developed for other scenarios provide a useful starting point for future work.
